# The Impact of Nutritional and Lifestyle Changes on Body Weight, Body Composition and Cardiometabolic Risk Factors in Children and Adolescents during the Pandemic of COVID-19: A Systematic Review

**DOI:** 10.3390/children8121130

**Published:** 2021-12-04

**Authors:** Kalliopi Karatzi, Kalliopi-Anna Poulia, Emilia Papakonstantinou, Antonis Zampelas

**Affiliations:** Laboratory of Dietetics and Quality of Life, Department of Food Science and Human Nutrition, Agricultural University of Athens, Iera Odos 75, 11855 Athens, Greece; pkaratzi@aua.gr (K.K.); lpoulia@gmail.com (K.-A.P.); apapakonstantinou@gmail.com (E.P.)

**Keywords:** diet, lifestyle, body composition, cardiometabolic risk, children, adolescents, COVID-19

## Abstract

The coronavirus (COVID-19) pandemic and the measures taken by most countries to curb virus transmission, such as social distancing, distance learning, population, home confinement and disruption of all organized activities, has affected children and adolescents worldwide. The aim of this review was to assess the role of diet and lifestyle changes due to COVID-19 measures on body weight/composition and cardiometabolic risk factors in children and adolescents. An electronic search was conducted in PUBMED, COCHRANE, Google Scholar and SCOPUS databases up to 31 October 2021. 15 eligible studies were identified. According to the studies included in the analysis, COVID-19 measures seem to have had a negative impact on the diets and lifestyles of children and adolescents, with a consequent increase in body weight and central fat accumulation. On the other hand, the parental presence and control resulted in better glycaemic control in children with diabetes mellitus (DM) Type 1, but the effect of the pandemic in the glycaemic control of children with DM2 2 is controversial. Finally, diet and lifestyle changes had a differential impact on children’s hypertension prevalence. These findings point to the need for public policy measures to prevent obesity and its complications, to and improve diet and lifestyle during the continuing and yet unresolved COVID-19 epidemic.

## 1. Introduction

Coronavirus disease (COVID-19) caused by the severe acute respiratory syndrome coronavirus 2 (SARS-CoV-2) is one of the most serious pandemics worldwide that has infected and killed millions of people. To curb virus transmission, several preventive measures have been taken by most of the countries on all continents, such as the limitation of travelling, distance learning in all educational levels, tele-working, and total confinement of the general population at home. These measures, also known as lockdown, caused disruption of daily activities in all ages due to the need for social distancing to control the epidemiology of the disease [1].

Some of the major consequences of the pandemic measures, such as social distancing and the shutdown of live educational activities have affected lifestyle and dietary behaviors related to overall health in both children and their families. Interestingly it was suggested that the lockdown is responsible for the consumption of poor-quality food, such as ultra-processed, calorie-dense comfort food when compared to standard living conditions [2,3]. The abrupt interruption of organized physical activities such as sports are paralleled with increased sedentary habits and screen time due to the inability to perform social gathering, distance learning, cancellation of the function of recreation programs and playgrounds and confinement at home. These changes have also affected sleep quality and duration. More specifically, the cancelation of live participation in educational activities attenuated all unfavorable consequences, as school increases opportunities for structured and recreational physical activities, decreases screen time, reduces opportunities for snacking and enables earlier school-day bedtimes [4]. Therefore, children’s social distancing enhanced feelings of boredom and stress, increased snacking of energy-dense foods and emotional eating, and at the same time reduced the participation in structured physical activities, and increased sedentary time and inadequate or low quality sleep time [5]. The consequent disproportion between energy intake and energy spent inevitably resulted in a positive energy balance, increased fat disposition and weight gain [2,6].

Childhood obesity is another epidemic and it is considered as the most important health problem of the 21st century concerning 38 million children under the age of 5, and over 340 million children and adolescents aged 5–19 [7,8]. It has a multi-factorial origin with genes, perinatal factors, social setting background, obesogenic environment, and lifestyle habits/dietary preferences, physical activity, sedentary time and sleep duration playing the most important roles [6].

The measures implemented due to the COVID-19 pandemic have created an obesogenic environment in children with less physical activity, longer sedentary time, unhealthy and energy dense food choices and sleep disturbances, which has probably led to even higher childhood obesity prevalence [9]. In turn, childhood obesity amplifies the risk of cardiometabolic conditions such as hypertension, dyslipidemia, diabetes and cardiovascular disease (CVD) [10,11,12]. At the same time, healthy food choices and regular physical activity are also considered to play a significant role in health in the early stages of life, which can also lower the risk of developing non-communicable (NCDs) diseases in adulthood [8].

These unprecedented living conditions, with confinement at home, increased food consumption and sedentary time during the day, may have led to significant alterations in body composition and body weight, as well as in deterioration of cardiometabolic health. As the COVID-19 pandemic remains a resisting and unresolved health problem, it continues to affect children’s lives and overall health.

Therefore, the aim of this systematic review was to collect and critically evaluate all the available scientific work related to the interplay between diet and lifestyle changes and children’s body composition and cardiometabolic risk during the COVID-19 pandemic, in order to enable public health initiatives for the prevention of the negative consequences of obesity and cardiometabolic risk early in life.

## 2. Materials and Methods

The present review was registered and published by PROSPERO, under the title “Systematic review of the role of lifestyle and diet on body composition changes and cardiometabolic risk factors in children during the pandemic of COVID-19” (registration number: CRD42021279972). The present work was prepared and presented in accordance with the guidelines of the PRISMA statements.

### 2.1. Search Strategy

**Figure 1:**
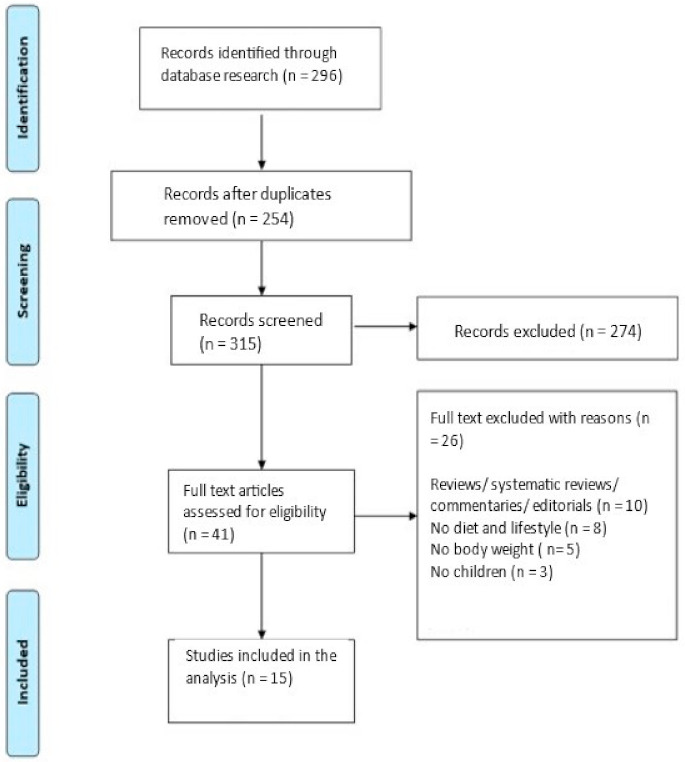
PRISMA flow chart of included studies.

A systematic search for eligible studies was performed through October 2021 by two separate reviewers on the PUBMED, COCHRANE, Google Scholar and SCOPUS databases using identical language, age, and date limits. The last day of searches in all mentioned databases was 31 October. Search keywords and medical subject headings (MESH terms) applied were: [(“diet” or “dietary” or “lifestyle”) and (“body composition” or “body weight” or “weight status” or “weight change” or “BMI” or “waist circumference” or “childhood obesity” or “children overweight/obese”) or (“cardiometabolic” or “cardiovascular” or “hyperlipidemia” or “dyslipidemia” or “hypertension” or “diabetes” or “metabolic syndrome”]. Studies were limited to the English language and human studies in children aged 0–18 years. Reference lists and related articles of included studies were also examined for additional relevant articles (Figure 1).

### 2.2. Eligibility Criteria

The studies that were decided to be included in the review were examining the possible role of diet and lifestyle changes on children’s body composition and cardiometabolic conditions. The inclusion criteria applied were peer-reviewed, epidemiological studies or clinical trials, English language, human studies, children 0–18 years of age, published from the year 2020 and onwards, with clear results regarding changes in body weight/body composition or parameters related to cardiometabolic health. The following exclusion criteria were applied: studies conducted in adults, animal studies, reviews, systematic reviews, meta-analyses, commentaries/letters and editorials.

### 2.3. Selection of Studies and Data Extraction

Two reviewers performed independent research in the databases mentioned before, for titles and abstracts of all the relative resources. The search results were then imported to EndNote Software for identification and removal of all duplicates. Afterwards the two reviewers removed articles according to eligibility criteria in order to determine the final number of studies to be included in the review. Discrepancies were resolved after discussion. After agreement, full text screening was carried out. Qualitative and quantitative data from all included articles were extracted by both reviewers. The extracted data included specific details for study design, population characteristics, assessment methods used and changes in diet, lifestyle, body composition or body weight and cardiometabolic conditions.

### 2.4. Risk of Bias

**Figure 2:**
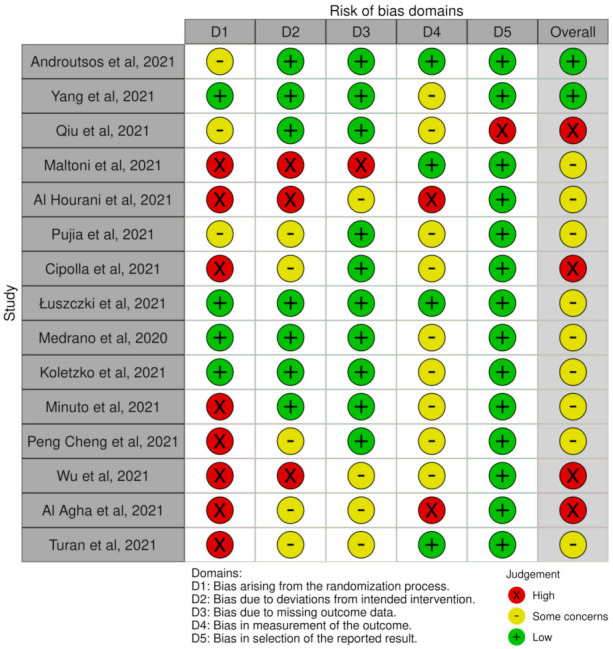
Studies with the representation of the Domains of risk of Bias.

**Figure 3:**
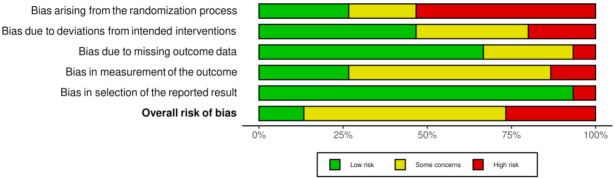
Overall presentation of the domains of risk among the studies.

A risk of bias tool was conducted with the Revised Cochrane risk-of-bias tool for randomized trials (RoB 2) [13]. The risk of bias covered the six domains of bias as described in the tool: election bias, performance bias, detection bias, attrition bias, reporting bias, and other bias. Relative plots were created for all the studies included in the analysis (Figure 2 and Figure 3).

## 3. Results

The initial search identified 296 references (Figure 1). After scanning for potential duplicates, 42 references were excluded. The remaining 254 references were screened according to title/abstract and reference lists, and relevant articles were screened. These procedures resulted in 315 references screened for relevance, out of which 274 were excluded. The remaining 41 articles underwent a full-text review based on inclusion and exclusion criteria. Further to this, 26 articles were excluded due to article type (editorials, commentaries or irrelevant reviews, *N* = 10) or due to inclusion/exclusion criteria, absence of diet and/or lifestyle outcomes (*N* = 8), absence of body composition/body weight outcomes (*N* = 5) adult population (*N* = 3). The final number of studies included in the present review is 15. These studies are summarized in Table 1 and Table 2.

### 3.1. Study Characteristics

The studies included in the present systematic review regarding the role of diet and lifestyle on body composition/body weight (Table 1) originate from two continents, four from Asia [15,16,18,29] and seven from Europe [14,17,19,20,21,22,23].The included studies for the role of diet/lifestyle on cardiometabolic risk factors (Table 2) originate from three continents, one from the Middle East [27], four from Asia [16,25,26,28] and two from Europe [24,30]. Interestingly the countries included present with large variation in their socioeconomic status having both developed countries like Germany and developing countries like Malaysia. The age of the participants ranged from 0–18 years and the study sample varied from very small i.e., 43 volunteers, [26] to very large i.e., 10,000 participants [15]. Regarding the study methodology, there were 12 cross-sectional studies, two retrospective studies and one longitudinal study.

According to the risk assessment of the studies, it has been noted that most of them are rather small and are based on convenience sampling, with no actual randomization, and including self-reported measurements of height and weight. Therefore, the selection bias and the accuracy of the answers are questionable (Figure 1). Under this spectrum, the overall risk evaluation in Figure 3 presents to be in greater extent either moderate or high. There were also studies mentioning measurements without presenting the relative results. This fact can be attributed to the eagerness to publish results for a new and unknown situation and at the same time the difficulty of performing assessments in person due to the lockdown and the limited access to the healthcare facilities.

### 3.2. Main Exposures

Changes in body weight/body composition were evaluated in relation to measures of change in diet and/or lifestyle (Table 1). All studies used self-reported data either using known validated questionnaires such as the Food Frequency Questionnaire (FFQ) and the International Physical Activity Questionnaire (IPAQ), or other questionnaires collected either by phone, live interviews, or online surveys. The primary outcomes were changes in body weight, Body Mass Index (BMI) and waist circumference, which were either measured (*N* = 5) [16,17,20,21,22] or self-reported (*N* = 5) [14,15,18,19,23]. Regarding the association of diet and/or lifestyle changes on cardiometabolic conditions, the primary outcome was blood pressure (*N* = 3) or glycemic control (*N* = 5).

### 3.3. Studies on the Association of Diet and/or Lifestyle Changes on Body Weight/Body Composition

The majority of studies assessed changes of both diet and lifestyle, except for two (one retrospective and one cross-sectional) which solely assessed parameters of physical activity and sedentary behavior [15,17]. Apart from one study that reported no results [16], all the other included studies reported increased consumption of fats and fast foods, as well as processed food, sweet and salty snacks and sugar sweetened beverages. Yet some studies also reported increased consumption of breakfast, fruit and vegetable intake and reduction in fruit juice and carbonated drinks consumption. Also, all studies shown in Table 1 reported a significant decrease in physical activity paralleled with a significant increase in sedentary and screen time, while two studies assessing sleep duration and sleep quality as part of the lifestyle assessment presented with controversial results [14,21]. Regarding the changes in body weight/body composition, eight out of the 10 studies included in the analysis and presented in Table 1 reported significant increases in body weight, BMI, waist circumference or body fat, which was evident even in the 59.7% of the study population [14,15,16,17,18,19,23]. According to Qiu et al., 28.1% of the children with normal BMI before the lockdown became overweight or obese, 42.4% of the overweight children became obese and 46.6% of the participants starting as normotensives presented abnormal BP levels at the end of the lockdown [16].

On the other hand, two studies reporting no changes in body weight/body composition measures during the pandemic also reported better adherence to the Mediterranean diet and reduced consumption of canned food, fast foods, snacks, fruit juices, sugar sweetened soft drinks and meat, resulting in an improved nutritional intake, an effect attributed to the increased family time and the time spent on cooking and meal preparation [21,22].

### 3.4. Studies on the Association of Diet and/or Lifestyle Changes on Cardiometabolic Risk Factors

Studies included in Table 2 presented outcomes regarding blood pressure and glycemic control, while no studies were retrieved for the association of diet and/or lifestyle changes during the pandemic with other cardiometabolic conditions. Regarding blood pressure, there are mixed results, as two studies reported increased blood pressure levels [16,27], while one study reported a relevant reduction [17], all paralleled with significant decreases in physical activity and increased time in sedentary behaviors. As regards glycaemic control studies, four studies were conducted solely in subjects with Type 1 diabetes mellitus (T1DM) [24,26,27,28] and one study included patients with both T1DM and type 2 diabetes mellitus (T2DM) [25]. Among them, two cross-sectional studies reported either increased plasma glucose levels by 18% [27] or increased glycosylated haemoglobin A1c (HbA1c) [28] levels by 0.32%, as a consequence of decreased physical activity, increased sedentary time and increased consumption of carbohydrates. Another cross-sectional study also reported elevated levels of sedentary behavior and decreased levels of physical activity together with altered meal frequency, mainly expressed as skipping breakfast. These dietary changes led to increased body weight and reduced HbA1c in prepubertal T1DM children due to closed parental control of the management of blood glucose levels. At the same time, it was connected with decreased body weight and elevated levels of HbA1c in children with T2DM and pubertal adolescents, signs of uncontrolled diabetes mellitus [25]. In another small cross-sectional study, the decrease of hypoglycemic episodes was reported, with no change observed in HbA1c, despite the decreased physical activity and increased number of daily snacks [26]. Finally, in a retrospective observational study, an improvement in glycemic control was also reported in children with T1DM despite the increase of sedentary activities and the limitation of physical activity, mainly due to the closer control of the food intake and the glucose measurements from their parents [24].

## 4. Discussion

Τhe impact of the COVID-19 pandemic on children has yet to be fully examined. There are justified concerns, confirmed by international organizations, that the health crisis that has emerged over the past two years may have severely affected the nutrition and lifestyles of children, with many unfavorable consequences with regard to childhood obesity and other cardiometabolic risks. Unfortunately, there are concerns that the COVID-19 pandemic threatens to reverse the hard-won progress achieved in the previous years in pediatric health.

The present systematic review aimed at gathering all existing knowledge from recently published articles regarding changes in diet and lifestyle during the COVID-19 pandemic and their effect on body weight and body composition, as well as cardiometabolic risk factors in children. These data may enable the design and implementation of public health preventive measures during periods of lockdown, particularly aiming at the enhancement of physical activity and healthy nutritional choices, especially when their duration is uncertain.

One of the main outcomes of the present systematic review is the fact that around the globe, similar dietary and lifestyle patterns were recorded [31,32]. The deterioration of food quality and the increment of food and energy quantity, alongside the mandatory confinement and the lower levels of physical activity and energy expenditure, combined to create an obesogenic environment for children. These changes were more profound in families with disadvantaged socioeconomic conditions, reflecting the economic impact of COVID-19 on nutrition and public health [33]. One of the main concerns is that these changes, which might be difficult to be reversed, can have short term and long-term negative effects on the overall health of the children in their childhoods and later on when they reach adulthood.

The tele-education and the lockdown resulted in increased screen time spent on computers and mobile phones, not as recreation but also as an obligatory responsibility. As a result, physical activity levels were compromised, and sedentary time increased significantly in children and adolescents [14,16,18,19,21,23]. It has already been reported by previous studies that the amount of time spent engaged in sedentary activities during childhood and adolescence predicts overweight and obesity in adulthood [34], posing a reasonable concern of the possible effects of this pandemic on the obesity epidemics that we will have to face in the following years.

A significant modification to sleep quality and overall sleeping habits was reported in many of the included studies. Some studies reported increased sleeping hours [18,27,32], or decreased sleep duration but increased sleep quality [22], or delayed sleeping time at night [28], affecting several aspects of the health of children and adolescents. Insufficient or interrupted sleep relates to mood swings and attention problems and poor mental health, situations made worse during the COVID-19 pandemic [35]. Sufficient sleeping time is essential in childhood. The American Academy of Sleep Medicine recommends that toddlers (aged 3–5 years) should get 10–13 h of sleep per day, school-going children (ages 6–12 years) should get 9–12 h per night, and adolescents should get 8-10 h nightly [36]. During the confinement, most of the studies reported an increase in sleep duration, explained by the absence of the obligation to attend school and other activities, which was not always achieved during the confinement. Moreover, the flexibility in waking and bedtime hours was the reason for increased sleep duration [35]. However, as circadian disturbances relate to weight gain during school breaks, these changes may have an impact in weight gain in children during COVID-19 [4].

All reported alterations observed regarding diet and lifestyle habits seem to have worsened the already existing problem of bad lifestyle choices and pediatric obesity. It is noteworthy that in the pre-COVID 19 era, more than 80% of children around the world were inactive, more than 60% did not meet recommended screentime guidelines, and nearly a half have a scarce adherence to the Mediterranean diet [37,38,39]. If the assumption that this picture will worsen during the pandemic is correct, it will negatively affect children ’s current and future health, and many scientists express their fear that the COVID-19 pandemic might shift towards an obesity pandemic. It is essential that more studies should be carried out with the primary aim of measuring the impact of this pandemic on the nutritional habits and health status of children and adolescents in different contexts. One of the main goals of these studies should be to assess whether unhealthy nutritional habits are maintained in the long term or even improved, and to objectively assess children’s nutritional parameters and compare them to the pre-pandemic era.

Another important aspect of the present review is the association of diet and lifestyle changes during the lockdown with cardiometabolic risk. Although the findings are controversial in some cases, there is limited evidence regarding worsening of glycemic control in children with T2DM and hypertension. On the contrary, the presence of parents and their active involvement in the management of T1DM resulted in a better management of children with T1DM. However, it is noteworthy that in most studies an increase in sweet and salty snacks, as well as sugar sweetened beverages, was observed, pointing out an increase in sugar and sodium intake, which makes us skeptical regarding possible underestimated unfavorable consequences due to COVID-19 on glycaemic control and hypertension. However, due to limited evidence, the heterogeneity of studies and the varied quality more well-designed studies examining the effects of COVID-19 on glycaemic control and hypertension are needed to draw credible conclusions.

Moreover, adolescence is known to be an overwhelming time, with physical, hormonal, and behavioral changes that were made even more profound during lockdown, increasing the prevalence of anxiety, boredom, and lack of motivation. Stress can negatively affect blood glucose and blood pressure management, alongside with the unbalanced nutrient and energy equilibrium, resulting in poor diabetes control and blood pressure in children with T2DM. Therefore, the lifestyle and stress management in younger patients with diabetes is undoubtedly of paramount importance to maintaining good diabetes self-care, lower cardiometabolic risk and overall better health.

## 5. Conclusions

The COVID-19 pandemic that emerged in the past two years is still uncontrolled, and measures to curb virus infection, such as confinement at home and social distancing, lead to a disruption of daily activities with yet unknown health consequences. The present systematic review is the first to our knowledge that gathered existent relevant studies highlighting the worsening of children and adolescents’ diet and lifestyle with unhealthy food choices, increased sedentary time, and decreased physical activities, which led to increased body weight and abdominal fat accumulation. Also, obesity, alongside with unhealthy diet and lifestyle with increased consumption of fat, sugar and sodium and increased time spent on sedentary activities are significant risk factors for cardiometabolic unfavorable consequences, such as diabetes and hypertension that are poorly studied or such as dyslipidemia and metabolic syndrome which are not studied at all, posing a great threat for an outburst of the existent obesity epidemic and its serious health implications. It is highly possible that the real consequences of COVID-19 on obesity and cardiometabolic conditions are understudied and possibly neglected, and therefore future public health initiatives are needed to assess and control obesity and its cardiometabolic complications in an early and timely fashion during this unprecedented public health crisis.

## Figures and Tables

**Table 1:** Characteristics of studies regarding the role of diet and lifestyle changes on body weight and body composition.

Identity	Country	Study Design	Participants (N)	Age Range (Years)	Diet Assessment	Lifestyle Assessment	Anthropometry and Body Composition Assessment	Outcome in Diet and Lifestyle	Outcome in Body Composition
Androutsos et al., 2021 [14]	Greece	Cross sectional Online survey	397	2–18	Questionnaire for the dietary habits of parents and children	Questionnaire on Sleep duration Screen time and Physical activity	Self-reported weight and height	↑ sleep duration ↑ screen time ↓ physical activity ↓ fast food ↑ fruit and vegetables ↑ breakfast	↑ Body weight in 35% of the participants
Yang et al., 2020 [15]	China	Retrospective study	10,082	16–18	N/A	Physical activity Questionnaire (IPAQ) long form	Self-reported weight and Height BMI	↓ physical activity frequency and intensity ↑ screen time	↑ BMI ↑ % Overweight/Obesity
Qiu et al., 2021 [16]	China	Cohort study	445	7–12	Questionnaire on diet	Questionnaires on lifestyle	Measured weight and Height	N/A	↑ % Overweight/Obesity
Maltoni et al., 2021 [17]	Italy	Cross sectional	51	10–18	Questionnaire on nutrition	Physical activity Questionnaire (IPAQ) sort form	Measured weight and Height Waist circumference	↑ Sedentary behavior ↓ Physical activity	↑ BMI ↑ Waist circumference ↑ Weight ↑ Weight/height ratio
Al Hourani et al., 2021 [18]	Jordan	Cross sectional	477	6–17	FFQ	Questionnaire on Screen time and Physical activity	Self-reported weight and height BMI for age Z scores	↑ Screen time ↑ Sedentary ↑Dietary intake	↑ BMI for age Z score ↑ Body Weight
Pujia et al., 2021 [19]	Italy	Cross sectional Online survey	439	5–14	Questionnaire on eating habits	Questionnaire on physical activity	Self-reported weight and height BMI	↑ Comfort food intake (chocolate, sweet snacks, and deserts ↑ pizza and bakery products ↓ sweetened beverages and candies ↑ sedentary lifestyle in adolescents	↑ Body Weight in 59.7% of the participants ↑ BMI
Cipolla et al., 2021 [20]	Italy	Cross sectional Telephone interview	64	8–18	Questionnaire on eating habits	Questionnaire on physical activity	Measured weight and height	↑ pizza and bakery products ↑ sedentary lifestyle ↑ Screen time	↑ BMI
Łuszczki et al., 2021 [21]	Poland	Cross sectional	1016	6–15	FFQ-6	Sleep quality and durationScreen timeSelf-reported physical activity	Measured weight and height BMI	↓ Sleep duration ↑ Sleep quality ↓ Physical activity ↑ Screen time ↓ fruit juices, carbonated sugar sweetened/diet drinks, meats ↓ canned food, fast f ood, snacks ↑ protein intake ↑ sweets	Non-significant changes
Medrano et al., 2020 [22]	Spain	Longitudinal cohort study (MUGI project)	291	8–16	Adherence to Mediterranean diet (KIDMED)	Physical Activity and screen time during leisure were assessed by “The Youth Activity Profile” questionnaire (YAP)	Measured Height and Weight BMI Body Composition (BIA) Waist circumference	↓ Physical activity ↑ Screen time ↑ KIDMED score	Non-significant changes
Koletzko et al., 2021 [23]	Germany	Cross sectional Online survey	1000 parents with at least 1 child<14 y living in the same household	0–14	Questionnaire on diet habits	Questionnaire on Physical activity	Self-reported weight gain	↑ Fruit and Vegetables ↑ Carbohydrates ↑ Salty/sweet snacks ↑ Soft drinks ↓ Physical activity ↑ cooking at home	↑ Body weight in 9% of the children

BMI: Body Mass Index, FFQ: Food Frequency Questionnaire, N/A: non applicable,↑increase,↓decrease,↑% increased percentage of patients,↓% decreased percentage of patients.

**Table 2:** Study characteristics regarding the role of diet and lifestyle changes on cardiometabolic conditions.

Identity	Country	Study Design	Participants (N)	Age Range (Years)	Diet Assessment	Lifestyle Assessment	Cardiometabolic Risk Factors	Outcome in Diet and Lifestyle	Outcome in Cardiometabolic Risk Factors
Qiu et al., 2021 [16]	China	Cohort study	445	7–12	Questionnaire on diet	Questionnaires on lifestyle	Blood pressure measured	N/A	↑% with Elevated Blood pressure
Maltoni et al., 2021 [17]	Italy	Cross sectional	51	10–18	Questionnaire on nutrition	Physical activity Questionnaire (IPAQ) sort form	Blood pressure	↑ Sedentary behavior ↓ Physical activity	↓% with Elevated blood pressure
Minuto et al., 2021 [24]	Italy	Retrospective observational cohort study	202 101 (0–18) with T1DM	6–39	N/A	Self-reported physical activity	GCM for glucose monitoring HbA1c	↓ Physical activity	Improved glycemic control
Peng Cheng et al., 2021 [25]	Malaysia	Cross sectional	123 (93 patients with T1DM and 30 with T2DM)	0–18	Standardized questionnaire for diet and lifestyle	Standardized questionnaire for lifestyle Physical activity assessment with PAQ-C for children and PAQ-A for older children;	HbA1c	↓ meal frequency (skipping breakfast) ↓ physical activity ↑ screen time ↑ sleep duration	↑ HbA1c in T2DM and pubertal adolescents ↓ HbA1c in pre-pubertal T1DM children ↑ Weight and BMI SDS in T1DM ↓ Weight and BMI SDS in T2DM
Wu et al., 2021 [26]	China	Observational study Telephone interview	43 with T1DM	0–18	Questionnaire for dietary intake	Questionnaire for, physical exercise, sleep habits and emotions	GCM for glucose monitoring HbA1c	↑ number of snacks, ↑ sleep duration ↑ time for diabetes management ↓ physical activity	No significant changes
Al Agha et al., 2021 [27]	Saudi Arabia	Cross sectional study	150 with T1DM	2–18	Questionnaire for dietary habits	Questionnaire for physical activity and mood	Blood pressure (systolic and diastolic) HbA1c CGM	↓ physical activity ↑ consumption of carbohydrates and fast food ↓ mood	↑ HbA1c ↑ BMI↑ Blood pressure
Turan et al., 2021 [28]	Turkey	Cross sectional	100 with T1DM	3–18	Questionnaire on snack and meal frequency, CHO consumption	Physical Activity Questionnaire-A (PAQ-A) or Physical Activity Questionnaire-C (PAQ-C)	HbA1c BMI	↑ consumption of carbohydrates Delayed sleep times	↑ HbA1c ↓ No of hypoglycaemic events

GCM: Glucose Continuous Monitoring, BMI: Body Mass Index, T1DM: Type 1 Diabetes Mellitus, T2DM: Type 2 Diabetes Mellitus, HbA1: Glycosylated Hemoglobin, N/A: non applicable, CHO carbohydrates,↑increase,↓decrease.↑% increased percentage of patients,↓% decreased percentage of patients.

## Data Availability

Not applicable.
